# The Influence of Green Brand Affect on Green Purchase Intentions: The Mediation Effects of Green Brand Associations and Green Brand Attitude

**DOI:** 10.3390/ijerph17114089

**Published:** 2020-06-08

**Authors:** Yu-Shan Chen, Tai-Wei Chang, Hung-Xin Li, Ying-Rong Chen

**Affiliations:** 1 Department of Business Administration, National Taipei University, New Taipei City 237, Taiwan; s810631103@gm.ntpu.edu.tw (H.-X.L.); cyr@ey.gov.tw (Y.-R.C.); 2 Graduate School of Resources Management and Decision Science, National Defense University, Taipei 112, Taiwan; allain1105@yahoo.com.tw

**Keywords:** sustainable consumption, green brand affect, green purchase intentions, green brand associations, green brand attitude

## Abstract

This study investigates the impact of green brand affect on green purchase intentions and explores the mediation effects of green brand attitude and green brand associations by means of the structural equation model (SEM). There is no previous literature discussing the relationship between brand affect and purchase intentions from the perspective of green marketing. Therefore, this article establishes a green purchase intention framework to fill in the research gap. The research object of this study focuses on Taiwanese consumers who have the purchase experience of information and electronics products in Taiwan. A total of 1000 consumers were randomly selected and 365 valid responses were received. In addition, this research conducted an empirical study using a questionnaire survey and structural equation model (SEM) to verify the research framework. The results show that green brand affect has no direct influence on green purchase intentions. Besides, this study indicates that green brand associations and green brand attitude fully mediate the relationship between green brand affect and green purchase intentions. It implies that green brand affect indirectly influences green purchase intentions via green brand attitude and green brand associations. While companies tend to raise their customers’ green purchase intentions, they need to increase their green brand affect, green brand associations, and green brand attitude.

## 1. Introduction

Sustainable consumption involves the consumption of services and products in a way that minimizes environmental impact and meets consumer needs for both present and future generations [1,2]. Sustainable consumption can be used to enhance sustainable performance, meet consumer needs, improve quality of life, enhance resource efficiency, increase the use of renewable energy, minimize waste, and reduce carbon emissions [3,4]. Consequently, sustainable consumption, which can help us to attain sustainable development goals (SDGs), plays a more imperative role in the market [5,6]. Sustainable consumption has become an important topic for companies in an era where consumer environmentalism is prevalent. The popularity of sustainable consumption in the market is an opportunity for green branding. This study is mainly from the perspective of green brands. We found that previous studies paid less attention to the relationship between sustainable consumption and green brands. Although there are some studies on green brand equity [7], there is no previous research on green brands in the field of sustainable consumption. In order to fill this research gap, this research proposes a new concept, ‘green brand affect’, and refers to Chaudhuri and Holbrook [8,9] to define ‘green brand affect’ as “consumers’ positive emotional response towards a brand in consequence of the brand’s environmental performance”. This study forms an integral framework about green purchase intentions. This study investigates the impact of green brand affect on green purchase intentions, and explores the mediation effects of green brand association and green brand attitude.

Green products are defined as products that would not damage the environment [7,10,11]. Green products are sustainable products designed to minimize their environmental impact throughout their entire life-cycle. The goals of the development of green products is to decrease waste, reduce carbon emissions, and maximize resource efficiency. Moreover, green brands refer to brands that can provide sustainable benefits over other brands [12]. Green brands are those brands that consumers associate with environmental sustainability. In addition, green brands appeal to consumers who care about environmental protection. Since the increasing recognition and awareness of environmentalism in the market, the positioning of green branding strategies is to build up a unique sustainable image in the targeted customers, in order to meet their green claims. Since more and more consumers are willing to give priority to greenness [13], by developing positive emotional responses as the basis for green brand influence, brand differentiation and generating green purchase intentions are crucial to green brand strategy.

Consumers gain from brands an opportunity to build and protract their identity and achieve a feeling of pleasure [14]. When consumers have a positive brand affect towards a particular brand, they will have a positive evaluation of the brand, thereby increasing loyalty and trust in the brand. Conversely, when consumers have a negative brand affect towards a particular brand, consumers will dislike the brand and generate a negative evaluation [15]. Matzler, Bidmon, and Grabner-Krauter [16] argue that brand affect can be used to observe the process of consumers buying the brand. Positive brand affect imples that the higher the potential for a brand to bring pleasure, the greater its potential to trigger a positive emotional response from consumers, which can make consumers more likely to purchase the brand’s products or services [17]. Therefore, positive emotions include comfort, satisfaction, security, fun, warmth, and familiarity; emotional commitments include unique emotions, identification, a special sense of the product, and a pleasant brand experience [17].

Brand strategies are important means to gain competitive advantage [18]. According to previous research, ‘brand affect’ plays an extremely important role in consumer behavior and purchasing decisions, confirming that emotion can be used as the main indicator for predicting consumer behavior [19]. Wright [20] also proposed the “emotion recommendation hypothesis”, which shows that consumers usually tend not to make decisions based on the information of specific attributes of the brand, but choose the brand where they feel the most positive emotions themselves. Brand affect can also be viewed as a comprehensive evaluation of consumer preferences or dislikes [21,22]. Recently, a great number of valuable frameworks have been constructed in the branding literature, inclusive of green brand affect [23], green brand associations [24,25], green brand attitude [24,26,27], and green purchase intentions [28,29,30,31,32,33].

Although previous literature widely explores the relevant topics about sustainable consumption, no research discusses sustainable consumption from the angle of green branding. Hence, this study would like to identify and fill the research gaps. Since green purchase intentions positively relate to sustainable consumption, this study focuses on the issue of green purchase intentions. This study proposes a new construct, green brand affect, and explores its influence on green purchase intentions, as proposed by Chen and Chang [28]. Additionally, this study also analyzes the mediation effects of green brand associations and green brand attitude. This study establishes a complete framework to examine the consequences of green brand affect on green purchase intentions in the field of sustainable consumption. Green purchase intentions are crucial for sustainable consumption in the era of prevalent environmentalism. This research develops a research framework which is beneficial for sustainable consumption by increasing green purchase intentions via its three determinants: green brand affect, green brand associations, and green brand attitude.

## 2. Literature Review and Hypothesis Development

**Figure 1:**
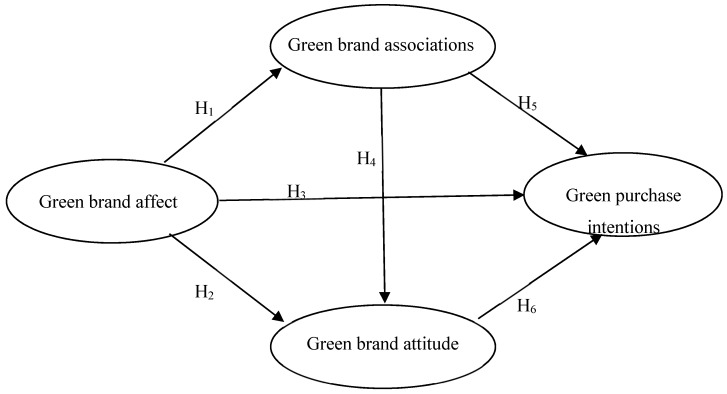
Research framework.

This study asserts that green brand affect positively influences green purchase intentions, while green brand associations and green brand attitude mediate the positive relationship between green brand affect and green purchase intentions. The antecedent of the research framework is green brand affect and the consequent is green purchase intentions, while green brand associations and green brand attitude are two partial mediators in this study. The research framework is shown in Figure 1.

### 2.1. The Positive Effect of Green Brand Affect on Green Brand Associations

“Brand association” is labeled as an important component of brand equity and it stands for consumer knowledge associated with a particular brand [34]. At present, brand associations play a critical role because of the strategic importance of brand communication in the intense branding context [21]. Although sustainable consumption has become a popular issue [35,36,37], little prior research focuses on green branding. Although green perceived value of product attributes has been explored [28], the role of green branding is still not systematically analyzed. With the increasing importance of sustainable consumption in the market, green branding provides an effective tool for the implementation of sustainable consumption. The motivation of this study is to analyze the influences of the key determinants of green branding on green purchase intentions.

Green brand associations are established based on delivering green product attributes related to the sustainability of the brand [38]. Keller [39] argues that brand associations can be characterized by three aspects: strength, favorability, and uniqueness. This study quoted the definition of Chen and Chang [24] to define ‘green brand associations’ as the extent to which consumers know about the green brands, and how they feel about and evaluate the green brand. Functional green branding has its limitations because a product’s environmental impact cannot generally reveal individual benefits to the buyer [7]. Green perceived value might be insufficient as a motivating factor for green purchase intentions [28].

A brand that can make consumers feel happy, excited, or cheerful will cause consumers to have more purchase behaviors for the brand and a higher loyalty attitude [8]. Green brands can evoke the green brand associations of targeted customers by providing them with green environmental information about the attributes of green products. A strong and effective green brand association can be obtained through the given emotional benefits that green brands actively evoke [38]. Green brands can associate green brands with the emotion of a customer to trigger a positive response to the brand after the target customer uses a certain brand. Past research indicates that brand affect can be used as the main indicator for predicting consumer behaviors [19]. This research proposes a new concept, green brand affect, and refers to Chaudhuri and Holbrook [8,9] to define ‘green brand affect’ as “consumers’ positive emotional response towards a brand in consequence of the brand’s environmental performance”. On the basis of the above statement, this study argues that green brand affect will have a positive impact on green brand associations, and suggest the following hypotheses:

**Hypothesis 1** **(H1).** *Green brand affect positively influences green brand associations.*

### 2.2. The Positive Effect of Green Brand Affect on Green Brand Attitude

Many companies are struggling to invest resources to create long-term relationships with customers [40,41]. It is a creative idea that brands have human characteristics which express consumer identity and fit with consumer self-concept [42,43]. Brand affect plays an important role in consumer purchasing decisions, and it is also an important predictor of consumer behaviors [19]. Besides, brand affect is the emotional factor that consumers have on the brand based on the recognition of the brand. Gobe [44] indicates that the effective strategy for brands to touch consumers is to create brand emotional attachment for consumers. Pawle and Cooper [45] assert that firms must communicate with consumers from the emotional side and build up amazing feelings for their consumers to maintain a long-term competitive advantage in the market. Brand affect represents the degree of consumer love for a specific brand. Only when consumers like a particular brand, will they be willing to buy again [46,47,48]. This research applies the work of Chen et al. [26] to define ‘green brand attitude’ as consumers’ attitude toward their overall evaluation of a brand’s green performance. In the context of environmentalism, the strength of brand attitude depends on the consumer’s evaluation of the brand’s strengths and weaknesses, and the level of positive evaluation of the brand [49]. This study argues green brand affect would positively affect green brand attitude, and we propose the following hypothesis:

**Hypothesis 2** **(H2).** *Green brand affect positively influences green brand attitude.*

### 2.3. The Positive Effect of Green Brand Affect on Green Purchase Intentions

Brand affect is one of the types of consumer associations with brands [16]. Moorman, Zaltman and Deshpande [50], and Morgan and Hunt [46], define brand affect as a brand’s ability to trigger a positive response from general consumers. In other words, the emotional feelings of consumers toward a certain brand include excitement, happiness, satisfaction, and so on. It can be viewed as a positive emotional commitment related to the brand’s sensory and emotional connections [17]. Russell [51,52,53] proposes facets for measuring brand emotions, namely, pleasantness–unpleasantness, and aroma–quietness on the polarization of emotional space. When consumers use a particular brand of products or services, they develop an evaluation of the brand’s likes or dislikes. Therefore, ‘brand affect’ refers to the emotional factors that consumers have when they recognize a brand. This emotion is the subjective feeling of a consumer after using a brand’s product. We can use brand emotions to observe the process of consumers buying the brand. A positive brand affect means that, when a brand has a higher potential to bring pleasure to customers, it has a greater potential to trigger a positive emotional response from consumers, making consumers more likely to buy the products or services of the brand [16]. It is a psychological phenomenon and is usually accompanied by emotions and moods. A brand that can make consumers feel happy or full of emotions will cause consumers to have more buying behaviors for the brand [8], which represents the consumer’s love for the brand. Only when consumers like a particular brand will they further generate satisfaction with the brand and the willingness to buy it again [46,47,48]. Brand affect is a very important key factor in the process of consumer purchase decision-making. Consumers’ purchase behaviors of green products will be influenced by their perceptions and attitudes [29]. Wright’s [20] proposed “impact referral” indicates that brand affect is in the step of the brand selection process. Most likely, consumers would ignore product information, but choose the brand that they think is the most positive. When a brand makes consumers feel happy, joyful, or full of emotions, it will cause consumers to buy more and become more loyal towards the brand [8]. It is an important indicator to predict consumer behavior [19], because consumers are full of emotions about brands, allowing brands to extend beyond simple repeat purchases [54,55,56].

We cite the definition from Chen and Chang [28], that ‘green purchase intentions’ is the possibility of consumers buying specific products or brands due to their environmental needs. In the context of environmental management, the above-stated views imply that green brand affect is positively associated with green purchase intentions, and we proposed the following hypothesis:

**Hypothesis 3** **(H3).** *Green brand affect positively influences green purchase intentions.*

### 2.4. The Positive Effect of Green Brand Associations on Green Brand Attitude

Associations refer to receivers’ fantasies and memories aroused by stimuli [57]. Brand cognitions could be positive or negative [58,59], so brand associations strongly relate to brand cognitions [60]. Furthermore, brand associations are part of brand cognitions, since they are connected with brand stimulus-based thoughts. Positive cognitions or thoughts regarding a brand lead to more favorable brand attitudes for the brand [61,62]. Positive cognitions have a positive effect on the development of positive brand attitude, while negative cognitions decrease the development of positive brand attitude [59]. Brand associations can evoke feelings and emotional responses [62,63,64]. Besides, if consumers have positive brand cognitions and perceive familiarity with the brand, a positive brand attitude may arise, leading to a positive affective reaction [65,66]. Prior research argues that positive brand associations positively impact brand attitude [66]. Therefore, green brand associations positively influence green brand attitude, which leads to our hypothesis:

**Hypothesis 4** **(H4).** *Green brand associations positively influence green brand attitude.*

### 2.5. The Positive Effect of Green Brand Associations on Green Purchase Intentions

The concept of brand associations was first proposed by Anderson [67]. Anderson [67] believes that brand association is a complex and comprehensive long-term memory model. Brand associations are linked in memory to a brand, based on a consumer’s frequent experiences with the specific brand [34]. Based on Keller’s [21] study, brand associations constitute consumers’ image of a particular brand, that is, their perception of the brand. Consequently, the brand association stored in the consumer’s memory can change the brand’s image [21]. Because brand associations allow firms to differentiate their brands in the market, they can influence consumers’ purchasing decisions. In addition, brand associations give consumers a reason to purchase products by influencing their attitudes and perceptions. Keller [21] categorizes brand associations into three dimensions: attributes, benefits, and attitudes. While brand attitudes are the consumers’ overall evaluations of the brand, benefits are the consumers’ perceived values attaching to the brand attributes. Besides, brand associations provide the basis for firms in positioning their brands. Many brand associations relate to product attributes or customer benefits that give a specific reason to purchase the brand’s products [34]. Marketers are interested in the strength of brand associations, because those associations can directly or indirectly affect consumers’ willingness to buy [68]. As a result, brand associations can influence consumers’ thinking while purchasing by conveying brand credibility and, moreover, establishing the brand’s reputation. Therefore, this article asserts that green brand associations have a positive effect on green purchase intentions, which leads to the following hypothesis:

**Hypothesis 5** **(H5).** *Green brand associations positively influence green purchase intentions.*

### 2.6. The Positive Effect of Green Brand Attitude on Green Purchase Intentions

Attitudes are defined as a person’s internal enduring evaluation of an object, issue, person, brand, product, or action [69,70]. Attitudes are often considered relatively stable and are an enduring tendency of consumers to behave in a specific way [71]. Hence, attitudes are useful predictors of consumers’ behaviors towards a product or brand [72]. Attitudes toward a brand refer to an inclination to respond in a favorable or unfavorable manner to a particular brand [73]. Purchase intentions are defined as the tendency to buy a certain brand or product [74]. Purchase intentions can be applied to measure how likely consumers would buy a specific product [73]. Attitudes play an important role in influencing consumers’ purchase intentions, which can be found in previous research [75,76]. As a result, we can find that the emotional factors of products can influence brand attitude and purchase intentions. It can be seen that brand attitudes reflect consumers’ evaluation of brands [42]. When you have a positive attitude towards a brand, you may increase your chances of adopting a brand [77]. Conversely, consumers who have a negative attitude towards a brand may reduce their chances of adopting the brand and destroy good relationships between them. Since previous research indicates that brand attitude positively influences purchase intentions [73,75,76], this research posits that green brand attitude positively relates to green purchase intentions and implies the following hypothesis:

**Hypothesis 6** **(H6).** *Green brand attitude positively influences green purchase intentions.*

## 3. Methodology and Measurement

### 3.1. Data Collection and Sample

The object of this study is concentrated on Taiwanese consumers who have experience in purchasing information and electronic products. We utilize questionnaires to examine hypotheses and research frameworks. The unit of analysis in this article is consumer level. The questionnaire was randomly sent to consumers who had a lot of experience in purchasing information and electronic products in Taiwan. We asked the respondents to answer the questionnaire items in relation to their recent purchases. Information and electronic products have to meet the international environmental regulations, such as the Paris Agreement, Restriction of the Use of Certain Hazardous Substances in EEE (RoHS) Directive, Waste Electronics and Electrical Equipment (WEEE) Directive, Energy Using Product (EuP) Directive, and Integrated Product Policy (IPP) Directive. Due to the popularity of green consumerism, manufacturers need to develop and market their information and electronic products so that they can satisfy their customers’ environmental needs [1,78]. This article refers to prior literature to design questionnaire items. The original questionnaire items were written in English, and they were later interpreted into Chinese by Taiwanese bilingual experts in the field of sustainable development. To reduce cultural prejudice and ensure the validity of the questionnaire, the Chinese version was eventually translated back to English by two other experts. They also have bilingual skills and specialty in the field of sustainable development in Taiwan. Such translation results can reduce any possibility of misunderstanding caused by translation. These back-translated questionnaire items are similar to the original English version. 

Before sending the questionnaire to the respondents, we asked six scholars to correct the questionnaire in the first pretest. Then, we randomly sent the questionnaires to ten consumers with information and electronic product purchase experience, asking them to fill out the questionnaire and revise the ambiguity in terms, meanings and problems in the second pretest. To ensure the content validity of the study, two rounds of pretests were conducted. After two pretests, we randomly selected samples from the “2018 Taiwan Yellow Book”. In order to have a high response rate, the research assistant randomly informed individual consumers with information and electronic product purchase experience. The electronic products we considered here include electric desk lamps, solar energy electric devices, environmentally friendly phones, electronic toothbrushes, energy-saving computers, electric fans, etc. Through observing the actual consumption experience, we made the respondents understand the research concept. Before sending the questionnaire, we carefully explained to the interviewees the objectives of the study and the contents of the questionnaire. Finally, we asked the interviewees to send back the completed questionnaires via email within two weeks. We wanted to ensure the acceptability of the survey content. Companies selling information and electronic products must comply with international environmental regulations, namely the Paris Agreement, Restriction of the Use of Certain Hazardous Substances in EEE (RoHS) Directive, Waste Electronics and Electrical Equipment (WEEE) Directive, Energy Using Product (EuP) Directive, and Integrated Product Policy (IPP) Directive. Therefore, consumers can purchase information and electronic products that meet their environmental protection requirements. A total of 1000 consumers were randomly requested to partake in the survey. Finally, 365 valid responses were received (a response rate of 36.5%). The results show that the sample of this study is comprised of 65.2% (238) university students. Furthermore, 72.1% (263) are 21–30 years old, 79.7% (291) earn under NT $400,000 annualy, and the majority are women (see Table 1).

### 3.2. The Measurement of the Constructs

All items were measured using the 7-point Likert scale from 1 (strongly disagree) to 7 (strongly agree). This research requires each respondent to identify specific information and electronic products of a given brand, to indicate that which gave them the best experience. Next, each respondent was asked to focus on the questionnaires relating to their preferred brand. We report more information on the questionnaires in Appendix A Table A1 and describe the definition and measurement of this study in the following:

Green brand affect. This research proposes a new concept, ‘green brand affect’, and refers to Chaudhuri and Holbrook [8,9] to define it as “consumers’ positive emotional response towards a brand in consequence of the brand’s environmental performance”. This paper refers to Chaudhuri and Holbrook [8] to measure green brand affect. The measurement of ‘green brand affect’ includes three items reported in Appendix A.

Green brand associations. This article adopts the definition of “green brand associations” by Chen and Chang [24], which is how consumers understand green brands, and how they feel and evaluate them. This article cites Chen and Chang [24] to measure green brand associations. The measurement of ‘green brand associations’ includes three items reported in Appendix A.

Green brand attitude. Chen et al. [26] define ‘green brand attitude’ as consumers’ attitude toward their overall evaluation of a brand’s green performance. This paper uses the measurement of green brand attitude developed by Chen et al. [26] and there are three items reported in Appendix A.

Green purchase intentions. The construct, green purchase intentions, is defined by Chen and Chang [28] as the likelihood that consumers would purchase given products or brands according to their environmental needs. In Chen and Chang’s study [28], the measurement of ‘green purchase intentions’ contains three items reported in Appendix A.

## 4. Empirical Results

This study uses the structural equation model (SEM) software, AMOS 26.0 (IBM, Armonk, NY, US), to verify the research framework and obtain the empirical results by means of the method of maximum likelihood estimation (MLE). This study used a goodness-of-fit test (Chi-square test) to verify that the data on the twelve items and the four constructs in the questionnaire were normal. We tested the null hypothesis, and the results proved that the four constructs and the twelve items observed in the sample all follow a normal distribution. In this study, the normal distribution is divided into ten regions, so the probability of each region is 0.1. This research computes the sixteen Chi-squared values for the twelve items and the four constructs, and all sixteen Chi-squared values (degree of freedom = 7, α = 0.05) are smaller than 14.0671. The results show that the measurement of this article follows the data normality. The results of the SEM analysis are shown below. 

### 4.1. The Results of the Measurement Model

The data such as means, standard deviations, and correlation matrices are shown in Table 2. We can see that there are positive correlations among green brand affect, green brand associations, green brand attitude, and green purchase intentions.

The exploratory factor analysis of the four constructs is shown in Table 3. We can classify each construct into one individual factor in Table 3.

This article cites existing research to design the questionnaire items. The study conducted two pretests on the revised questionnaire before mailing it to the interviewees. Thus, in terms of content validity, the metric of this study is acceptable. Moreover, we use Harman’s one-factor test to verify the existence of common method differences (CMV) [79]. Based on the result of the exploratory factor analysis, there are four factors. If there is a large amount of common method variance (CMV), a single factor will appear in the exploratory factor analysis, or a general factor will account for most of the covariance [80]. Rather than a single factor, there are four unique factors’ eigenvalues larger than 1.0. In addition, these four factors together accounted for 81% of the total variance, and the first (largest) factor does not address a majority of the variance (31%). Thus, no general factor is apparent. Based on the above two criteria, there is no common method variance (CMV) in this research.

There are some ways to examine the reliability and validity of the measure. First, one method to measure reliability is to check the load of a single item for each construct. Regarding the quality of the measurement model, the loadings (λ) of all items of the four constructs listed in Table 4 are significant. Secondly, Cronbach’s α is another indicator of reliability. The Cronbach’s α of these constructs are shown in Table 4. Generally, the minimum requirement for Cronbach’s α coefficient is 0.7 [81]. In Table 4, the Cronbach’s α coefficient of “green brand affect” is 0.917; that of “green brand associations” is 0.899; that of “green brand attitude” is 0.926; and that of “green purchase intentions” is 0.943. Because the Cronbach’s α coefficients of all constructs are greater than 0.7, the measurement results of this study are acceptable in reliability.

Thirdly, we utilize Fornell and Larcker’s measure, average variance extracted (AVE), to estimate the discriminant validity of the measurement [82]. AVE measures the amount of change captured by its term corresponding to the amount of change caused by measurement errors. The square root of the structured AVE must be larger than each structure in the model, including the correlation with other structures, so as to meet the requirements of discriminant validity. For example, the square roots of the two AVEs of green brand affect and green brand associations in Table 4 are 0.887 and 0.865, which is greater than the correlation coefficient, 0.686, between them in Table 2. There is acceptable discriminant validity between the two constructs, green brand affect and green brand associations. The square roots of the AVE of all the constructs in Table 4 are greater than the correlations between all the constructs in Table 2. Fourthly, in Table 4, the results demonstrate that the AVEs of all variables were more than 0.5, which means that the convergent validity of the four constructs was acceptable. Based on above analysis, the results indicate that there was adequate reliability and validity for all variables used in this study.

### 4.2. The Results of the Full Model

**Figure 2:**
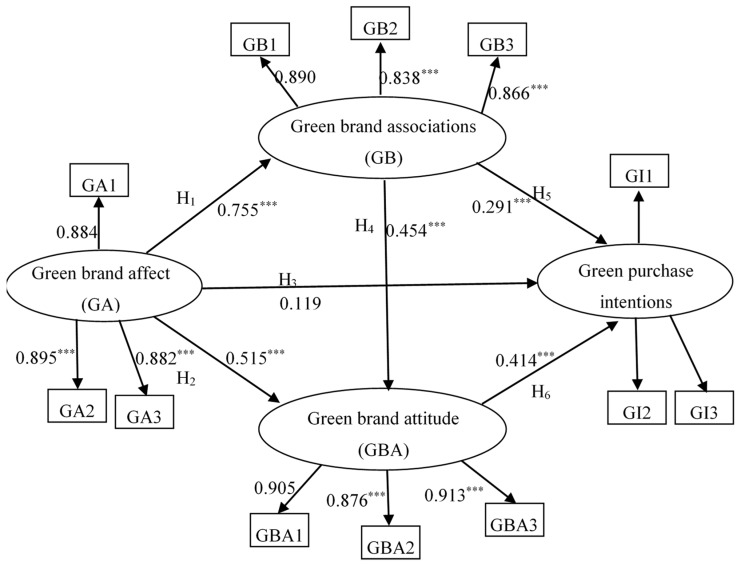
The result of the full model. Chi square/df = 2.224, GFI = 0.954, RMSEA = 0.058, IFI= 0.987, CFI = 0.986 Note: ^***^ *p* < 0.001.

In this research, structural equation modelling (SEM) is applied to test the hypotheses by using AMOS. The results show that GFI = 0.954, IFI = 0.987, RMSEA = 0.058 and CFI = 0.986, and the overall model fitness is acceptable. The coefficients of all paths are as follows: The coefficients of the H_1_, H_2_, H_4_, H_5_ and H_6_ paths are 0.755, 0.515, 0.454, 0.291, and 0.414, respectively, which are significant. Among the coefficients of the H_3_ path is 0.119, which is not significant. The residuals of the covariance are centered near 0 and relatively small. Figure 2 demonstrates the full model result.

Table 5 demonstrates the results that support H_1_, H_2_, H_4_, H_5_, and H_6_, but do not support H_3._ This study shows that (1) green brand affect positively influences green brand associations and green brand attitude; (2) green brand associations positively influence green brand attitude and green purchase intentions; and (3) green brand attitude positively influences green purchase intentions.

Based on the bootstrapping method of Taylor, MacKinnon and Tein [83], 5000 bootstrap samples are executed at a 95% confidence interval, and deviation correction percentile bootstrap and percentile bootstrap are performed to verify green brand affect (GA), green brand associations (GB), green brand attitude (GBA), and relationship with green purchase intentions (GI). There are three intermediary relationship models in Table 6: (1) point estimation = 0.256 (z = 2.589, z > 2.58, GA→GB→GI); (2) point estimation = 0.248 (z = 3.1, z < 3.29, GA→GBA→GI); (3) point estimation = 0.165 (z = 2.2, z > 1.96, GA→GB→GBA→GI), where, within a 95% confidence interval, each is indirect. There is no zero between the effect’s lower and upper limits [84]. Therefore, we can conclude that the three intermediary relationship models have achieved significant effects. Because green brand affect doesn’t significantly influence green purchase intentions, these three intermediary relationships belong to the full intermediary model. This study demonstrates that green brand associations and green brand attitude fully mediate the positive relationship between green brand affect and green purchase intentions. For that reason, companies should increase their customers’ green brand affect, green brand associations, and green brand attitude to enhance their customers’ green purchase intentions.

## 5. Discussion

For managers, effective ways of green branding are very critical for sustainable consumption, since green brand affect positively influences green brand associations and green brand attitude. There are three academic contributions in this study. Firstly, this study summarizes the notion of green branding to extend the literature on sustainable consumption and to raise green purchase intentions from the increase of green brand affect, green brand associations, and green brand attitude. Secondly, there is no research exploring the relationships among green brand affect, green brand associations, green brand attitude, and green purchase intentions. This study proves that green brand affect can positively influence green purchase intentions through green brand associations and green brand attitude, thereby filling the research gap. Thirdly, this study shows that the relationship between green brand affect and green purchase intention is fully mediated through green brand associations and green brand attitude. Increasing green brand affect, green brand associations, and green brand attitude leads to the increase of green purchase intentions. This article extends the field of green brand research to the field of sustainable consumption.

There are three practical contributions in this study. Firstly, the research results demonstrate that green brand affect is positively associated with green brand associations and green brand attitude, which are positively related to green purchase intentions. When companies enhance their consumers’ green brand affect, they have to raise both their consumers’ green brand associations and green brand attitude in order to further increase their consumers’ green purchase intentions. Secondly, in the sustainability era, it is worth developing capable retailers as an effective information channel between end users and the original brand manufacturers to increase green brand affect, green brand associations, and the green brand attitude of the end users in order to strengthen the green purchase intentions of the end users toward the manufacturers’ brands. Thirdly, because there exist full mediation effects of green brand associations and green brand attitude in this study, companies can improve the green brand associations and green brand attitude of their consumers to increase their consumers’ green purchase intentions. Although green brand affect can’t directly influence green purchase intentions, green brand affect can influence green purchase intentions indirectly via green brand associations and green brand attitude. Hence, if companies would like to increase their customers’ green purchase intentions, they need to raise their green brand affect, green brand associations and green brand attitude.

This research can help managers to develop a more effective green branding approach to stimulate sustainable consumption. Firms have to thoroughly recognize the sources of green purchase intentions, and to strengthen them. In other words, firms can raise their consumers’ green brand affect, green brand associations, and green brand attitude in order to increase their consumers’ green purchase intentions. The research object of this study focused on Taiwanese consumers who have the purchase experience of information and electronics products in Taiwan. Therefore, future research can focus on the purchasing experience of other products in other countries or regions and compare it with this research. We tested the hypotheses by using a questionnaire survey, only providing cross-sectional data, so it’s difficult to observe the dynamic change of green brand affect, green brand associations, green brand attitude, and green purchase intentions in the different periods. Due to this, future research can be developed in the direction of longitudinal research to learn the differences between green brand affect, green brand associations, green brand attitude and green purchase intentions in disparate periods. We have the intention that the results of this study can stimulate more future research, not only for researchers, but also for managers, practitioners and decision makers.

The first limitation of this study is the concentration on Taiwanese consumers who have experience in purchasing information and electronic products, so future research can focus on the purchase experience of other products. Speaking of the second limitation, the research context of this study is in Taiwan. Future research can be conducted in other countries and be compared with this study. Speaking of the third limitation, this study tests the hypotheses with a questionnaire survey, only providing cross-sectional data, so future research can set forth toward a longitudinal study to observe the dynamic change in the different stages. Finally, we hope that the research results are helpful to managers, researchers, practitioners, and policy makers, and contribute to future research as reference.

## 6. Conclusions

Green brand affect is a crucial determinant in the market. Prior research is not conclusive on how green purchase intentions can be enhanced and contained an integral way under the prevalence of sustainable consumption these days. Therefore, we stimulate green purchase intentions by developing a conceptual approach to green brands. This study designs a research framework to explore the impact of green brand affect on green purchase intentions and addresses the intermediary roles of green brand associations and green brand attitude. We find out that green brand associations and green brand attitude have full mediation effects on the positive relationship between green brand affect and green purchase intentions, as stated by the empirical results. Green brand affect is positively associated with green brand associations and green brand attitude, which positively influence green purchase intentions. Therefore, this research suggests that companies must strengthen their consumers’ green brand associations and green brand attitude when improving their consumers’ green brand affect, which can effectively increase their consumers’ willingness to buy green products. Because sustainable consumption has become more popular nowadays, companies need to utilize the green opportunities to market their products in order to increase their market share. The major challenge for firms is how to enhance green purchase intentions in the sustainable era. This study integrates the idea of green branding to propose a research model of green purchase intentions. Companies should actively differentiate and position their green products to obtain a higher market share in the green market.

The main purpose of this study is to discuss the relationship between green brand affect and green purchase intention, and to explore the mediating role of green brand associations and green brand attitude. Companies have to raise their green brand affect, green brand associations and green brand attitude to increase their consumers’ green purchase intentions. An effective way for managers to stimulate sustainable consumption is to enhance green brand affect, green brand associations, and green brand attitude, which result in increasing green purchase intentions to establish long-term relationships with their customers. Firms have to plan how to effectively allocate their resources in order to raise the key factors of green purchase intentions of their targeted consumers under the premise of limited resources. Both green brand associations and green brand attitude are positively related to green purchase intentions, so companies must enhance their consumers’ green brand associations and green brand attitude by improving their consumers’ green brand affect in the context of increased prevalence of sustainable consumption. Since information and electronic products have to comply with strict international environmental acts, such as the Paris Agreement, Restriction of the Use of Certain Hazardous Substances in EEE (RoHS) Directive, Waste Electronics and Electrical Equipment (WEEE) Directive, Energy Using Product (EuP) Directive, and Integrated Product Policy (IPP) Directive, and the popularity of green consumerism, information and electronic companies need to develop and market their information and electronic products so that they can meet their customers’ green needs. According to the results of this study, information and electronic companies should increase their green brand affect, green brand associations, and green brand attitude to enhance their green purchase intentions. 

## Figures and Tables

**Table 1:** Sample distribution by classification.

Classification		Frequency	Percent	Classification		Frequency	Percent
Education level	Graduate School (or above)	108	29.6%	Gender	Male	121	33.2%
	University (College)	238	65.2%		Female	244	66.8%
	High school (or below)	19	5.2%	Age	41 or above	12	3.2%
Annual income	1 million or more	9	2.5%		31–40	23	6.3%
	0.4–1 million	65	17.8%		21–30	263	72.1%
	Less than 0.4 million	291	79.7%		Below 20	67	18.4

Notes: (1) *N* = 365; (2) Annual income is denominated in New Taiwan Dollars.

**Table 2:** Means, standard deviations, and correlations of the constructs.

Constructs	Mean	Std. Dev.	1.	2.	3.	4
1. Green brand affect	5.424	0.9584	(0.887)			
2. Green brand associations	5.155	1.023	0.686 ^**^	(0.865)		
3. Green brand attitude	5.321	1.007	0.791 ^**^	0.764 ^**^	(0.898)	
4. Green purchase intentions	5.321	1.074	0.645 ^**^	0.674 ^**^	0.708 ^**^	(0.920)

Notes: ^**^ *p* < 0.01.

**Table 3:** Factor analysis of this study.

Constructs	Number of Items	Number of Factors	Accumulation Percentage of Explained Variance
Green brand affect	3	1	85.753%
Green brand associations	3	1	83.187%
Green brand attitude	3	1	87.133%
Green purchase intentions	3	1	89.715%

**Table 4:** The items’ loadings (λ) and the constructs’ Cronbach’s α coefficients and average variance extracted (AVE)s.

Constructs	Items	λ	Cronbach’s α	AVE	The Square Root of AVE
1. Green brand affect	GA1 GA2 GA3	0.884 0.895 ^***^ 0.882 ^***^	0.917	0.787	0.887
2. Green brand associations	GB1 GB2 GB3	0.890 0.838 ^***^ 0.866 ^***^	0.899	0.748	0.865
3. Green brand attitude	GBA1 GBA2 GBA3	0.905 0.876 ^***^ 0.913 ^***^	0.926	0.807	0.898
4. Green purchase intentions	GI1 GI2 GI3	0.929 0.912 ^***^ 0.918 ^***^	0.943	0.846	0.920

Note: ^***^ *p* <0.001.

**Table 5:** The results of the structural model.

Hypothesis	Proposed Effect	Path Coefficient	Results
H_1_. green brand affect positively influences green brand associations.	+	0.755 ^***^	H_1_ is supported
H_2_. green brand affect positively influences green brand attitude.	+	0.515 ^***^	H_2_ is supported
H_3_. green brand affect positively influences green purchase intentions.	+	0.119	H_3_ is not supported
H_4_. green brand associations positively influence green brand attitude.	+	0.454 ^***^	H_4_ is supported
H_5_. green brand associations positively influence green purchase intentions.	+	0.291 ^***^	H_5_ is supported
H_6_. green brand attitude positively influences green purchase intentions.	+	0.414 ^***^	H_6_ is supported

Note: ^***^ *p* < 0.001.

**Table 6:** The mediation results of this study.

	Point Estimation	Product of Coefficients		Bootstrapping			
				Bias-Corrected 95% CI		Percentile 95% CI	
		S.E.	Z	Lower	Upper	Lower	Upper
Indirect Effects							
(1) GA→GB→GI	0.256 *	0.099	2.586	0.069	0.457	0.062	0.450
(2) GA→ GBA→GI	0.248 **	0.080	3.1	0.110	0.436	0.098	0.412
(3) GA→ GB→GBA→GI	0.165 *	0.075	2.2	0.056	0.360	0.047	0.336
Direct Effects							
(4) GA→GI	0.138	0.121	1.140	−0.090	0.383	−0.083	0.392
Total Effects							
Total (1 + 2 + 3 + 4)	0.669 ***	0.068	9.838	0.674	0.941	0.671	0.939
Contrasts							
(1)—(2)	0.008	0.157	0.051	−0.303	0.312	−0.299	0.318
(2)—(3)	0.083	0.073	1.137	−0.041	0.257	−0.073	0.225
(3)—(1)	−0.083	0.073	1.137	−0.257	0.041	−0.225	0.073

Notes: (1) Standardized estimating of 5,000 bootstrap samples; (2) Contrasts of the two indirect effects; (3) Green brand affect (GA); Green brand associations (GB); Green brand attitude (GBA); Green purchase intentions (GI); (4) * Z > 1.96, ** Z > 2.58, ***Z > 3.29; (5) N = 365.

## Appendix A

**Table A1:** The information of the questionnaire.

Constructs	Items		Cronbach’s α	Resources
	Numbers	Content		
Green brand affect	GA1	The environmental friendliness of the brand makes you feel good.	0.917	Chaudhuri and Holbrook [8]
	GA2	the brand’s emphasis on environmental protection makes you feel good.		
	GA3	the brand’s environmental performance makes you happy.		
Green brand associations	GB1	The strength of the brand’s environmental features is outstanding.	0.899	Chen and Chang [24]
	GB2	the favorability of the brand’s environmental features is better than that of other brands.		
	GB3	the uniqueness of the brand’s environmental features is excellent.		
Green brand attitude	GBA1	You will like this brand more because the brand is environmentally friendly.	0.926	Chen et al. [26]
	GBA2	You will prefer the brand because of your concern for the environment.		
	GBA3	You agree that the brand can be more valuable because it is environmentally friendly.		
Green purchase intentions	GI1	You intend to buy the brand out of concern for the environment.	0.943	Chen and Chang [28]
	GI2	You bought the brand last because of its environmental performance.		
	GI3	In general, you are happy to buy the brand considering it is environmentally favorably.		
